# Changes in dietary intake of aspartic acid during and after intermittent fasting correlate with an improvement in fasting glucose in overweight individuals

**DOI:** 10.1111/1753-0407.13351

**Published:** 2023-01-09

**Authors:** Spyridon N. Karras, Theocharis Koufakis, Georgios Dimakopoulos, Djordje S. Popovic, Kalliopi Kotsa

**Affiliations:** ^1^ Division of Endocrinology and Metabolism, First Department of Internal Medicine Medical School, Aristotle University of Thessaloniki, AHEPA University Hospital Thessaloniki Greece; ^2^ BIOSTATS, Epirus Science and Technology Park Campus of the University of Ioannina Ioannina Greece; ^3^ Clinic for Endocrinology, Diabetes and Metabolic Disorders Clinical Centre of Vojvodina Novi Sad Serbia; ^4^ Medical Faculty University of Novi Sad Novi Sad Serbia

## Abstract

**Highlights**

- We investigated a potential correlation between amino acid intake and glycemic markers among individuals who practiced intermittent fasting and controls.
- Reduced aspartic acid intake during and after intermittent fasting presented a positive correlation with fasting glucose.
- The positive effects of intermittent fasting on glucose metabolism could be partially related to a decrease in the ingestion of specific amino acids.

To the editor,

1

Accumulating evidence suggests a positive impact of intermittent fasting (IF) on the glycemic profile.^1^, ^2^ Although the exact underlying mechanisms are still under investigation, it has been proposed that they could be related to upregulation of adaptive cellular responses, such as autophagy, mitochondrial function, and stress response pathways.^3^ Previous research has indicated that ingestion of specific amino acids can stimulate insulin and glucagon release.^4^, ^5^ However, prospective data on changes in amino acid intake and their impact on glucose levels during IF are currently unavailable. This study aimed to investigate a possible correlation between amino acid intake and glycemic markers among overweight, still metabolically healthy individuals who practiced IF.

## 2 METHODS

Fasting plasma glucose (FPG), fasting plasma insulin, homeostatic model assessment for insulin resistance (HOMA‐IR), anthropometry, and amino acid dietary intake were evaluated in 14 adults (mean age and body mass index [BMI] 46.3 years and 28.3 kg/m^2^, respectively) who practiced 16:8 time‐restricted eating (TRE). Measurements occurred at three time points: before TRE (baseline), at the end of the diet intervention (7 weeks), and 5 weeks after participants returned to their typical eating habits (12 weeks). The same parameters were evaluated in 29 individuals (mean age and BMI 49.9 years and 29.0 kg/m^2^, respectively) who followed Orthodox fasting (OF), a subset of the typical Mediterranean diet and served as controls. All participants had no history of chronic metabolic disease and did not receive supplements or medications. The amino acid composition of the diets was based on the analysis of 3‐day food records with the Food Processor Nutrition Analysis Software.^6^ Written informed consent was obtained from all included subjects, and the study protocol was approved by the responsible ethics committee.

## 3 RESULTS

**FIGURE 1:**
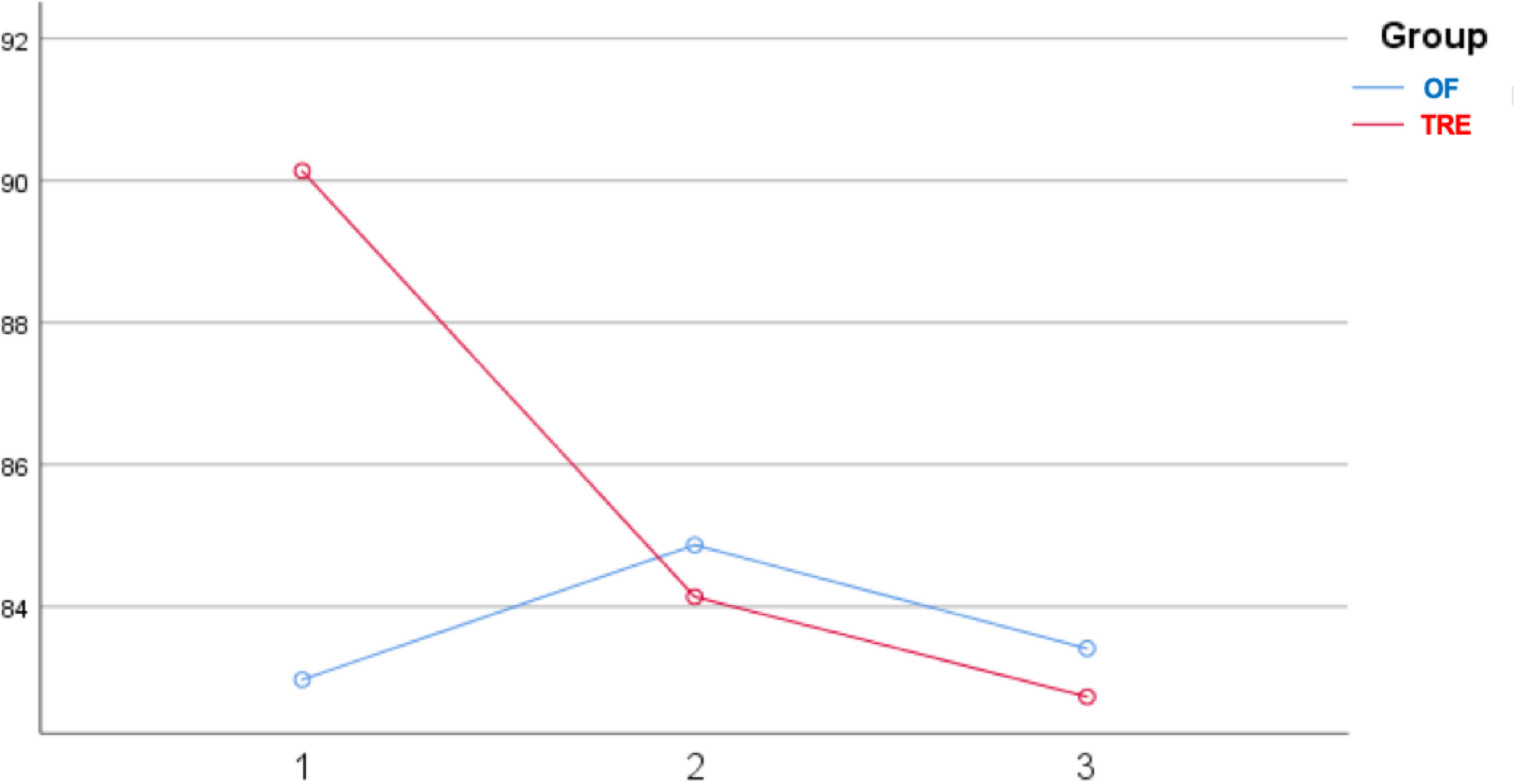
Changes in fasting glucose levels in the two groups during the study. OF, Orthodox fasting; TRE, time‐restricted eating. Vertical axis: fasting glucose (mg/dl); horizontal axis: time point

**FIGURE 2:**
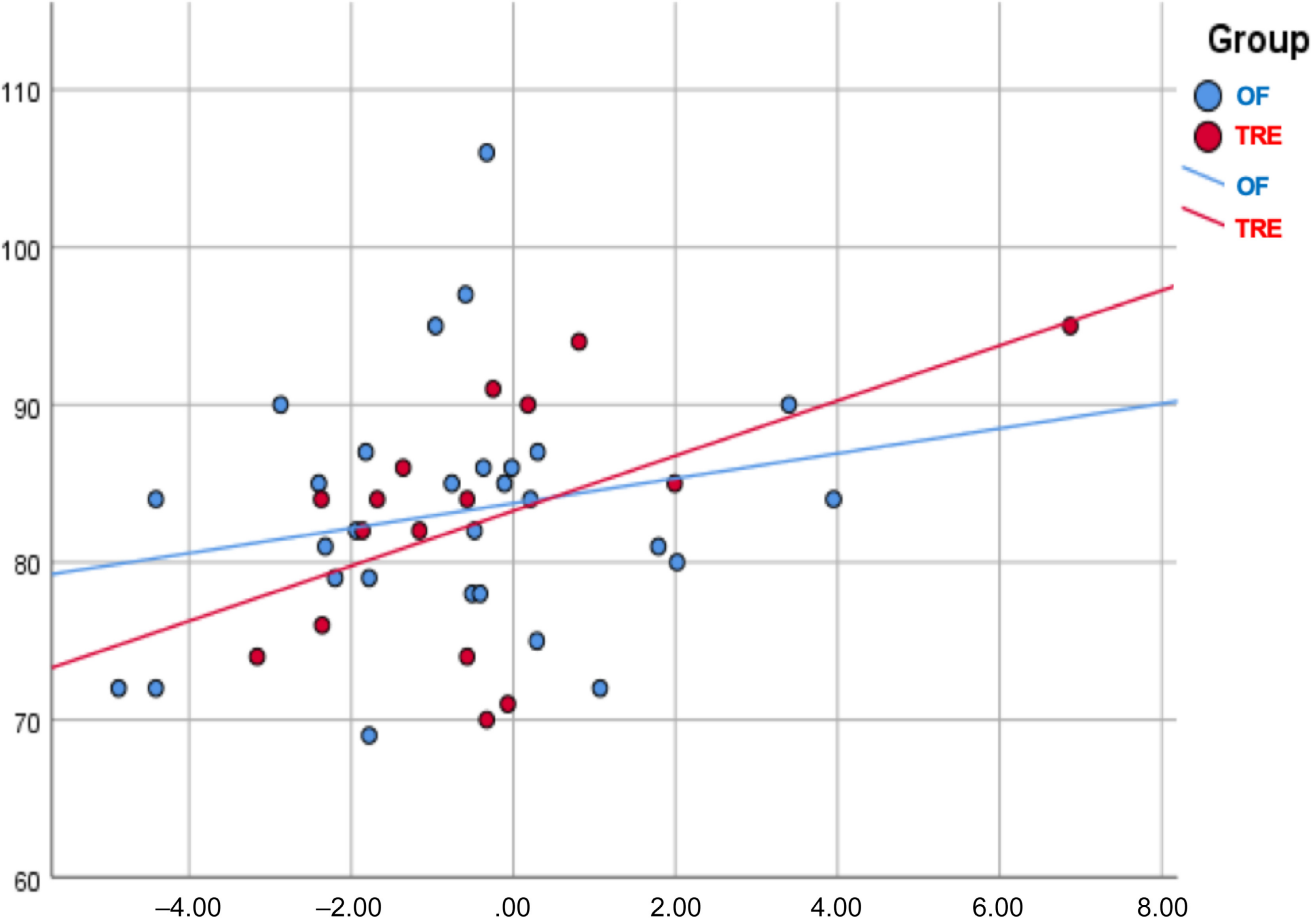
Correlation between aspartic acid intake and fasting glucose at 12 weeks in the two groups. OF, Orthodox fasting; TRE, time‐restricted eating. Vertical axis: fasting glucose (mg/dl) at 12 weeks; horizontal axis: aspartic acid intake (gr)

At baseline, participants in the two groups presented comparable characteristics in terms of anthropometric and glycemic markers. Repeated measures analysis of variance showed that both groups experienced a reduction in BMI after diets compared to baseline values (TRE: 28.3 ± 6.7 kg/m^2^ [baseline] vs. 27.5 ± 6.3 kg/m^2^ [7 weeks], *p* < .001 and OF: 29.0 ± 6.0 kg/m^2^ [baseline] vs. 28.2 ± 5.4 kg/m^2^ [7 weeks], *p* < .001). During the dietary intervention, a significant decrease in the daily intake of aspartic acid (8.77 vs. 5.90 g; *p* < .01), alanine (4.42 vs. 2.99 g; *p* < 0.01), and methionine (2.10 vs. 1.44 g; *p* < .01) was observed in the TRE group. Such differences were not documented in the control group for any of the amino acids examined. The TRE group had a lower intake of aspartic acid than the OF group at seven (*p* = .03) and 12 weeks (*p* < .01). In the TRE group, FPG presented a decreasing trend during the study: 90.14 mg/dl (baseline) vs. 84.50 mg/dl (7 weeks) vs. 83.29 mg/dl (12 weeks), with the difference significant between baseline and 12 weeks (*p* = 0.021) (Figure 1). In contrast, there were no significant fluctuations in the FPG values of the controls: 82.97 mg/dl (baseline) vs. 84.69 mg/dl (7 weeks) vs. 83.14 mg/dl (12 weeks) (*p* > .05 for all comparisons). No differences in FPG values were documented between the two groups at any time. General linear models for repeated measures showed that the change in aspartic acid intake presented a positive correlation with FPG values in the TRE group, suggesting that the greater the reduction from baseline to 12 weeks, the lower the expected FPG concentrations at the final time point (*p* = .039) (Figure 2). However, the significance diminished when BMI and body fat were included in the model (*p* = .049). In the TRE group, the effect of changes in alanine and methionine intake on FPG values at 12 weeks was found to be marginally nonsignificant (*p* = .052 and *p* = .054, respectively). No correlations were established between amino acid ingestion and insulin or HOMA‐IR values in either group.

## 4 COMMENT

To our knowledge, this pilot study is the first to assess the impact of amino acid intake on glucose homeostasis in people practicing IF. Our findings suggest that the positive effects of TRE on fasting glucose could be partially related to a decrease in the ingestion of specific amino acids. Lindgren et al have shown that oral administration of amino acid mixtures stimulates a stronger insulin secretory response compared to intravenous administration mediated by an increase in gastric inhibitory polypeptide concentrations, suggesting the existence of an incretin effect similar to that elicited by intestinal glucose absorption.^7^ However, plasma glucose levels generally remain unaffected by amino acid ingestion, possibly because of compensatory stimulation of gluconeogenesis.^5^ Zhou et al have recently shown that leucine restriction in mice led to reduced fat mass and improved glucose regulation, mediated by a decrease in lipid synthesis in adipose tissue, as well as improved insulin sensitivity in muscles.^8^ Short‐term dietary restriction of branched‐chain amino acids has been reported to improve postprandial insulin sensitivity through alterations in gut microbiome composition.^9^ It becomes evident that the full spectrum of pathways through which amino acids can influence glucose metabolism remains obscure. Animal research has suggested as possible mechanisms improvement in insulin production and peripheral insulin sensitivity related to decreased lipid synthesis in fat stores, favorable changes in incretin hormone equilibrium, and amelioration of gut dysbiosis.

Our findings should be interpreted in light of some limitations, such as the small sample size, the nonrandomized character of the study, and the fact that the estimation of amino acid intake was based on reported food consumption and not blood measurements. Therefore, the preliminary results reported in this letter should be tested in a larger study. Given the complexity of the factors that contribute to glucose homeostasis, more studies are needed to replicate these findings and elucidate the relevant mechanisms, especially in the context of specific dietary patterns such as IF.

## DISCLOSURE

The authors report that they have no conflict of interest relevant to the present work.

## References

[1] Karras SN , Koufakis T , Adamidou L , et al. Effects of Christian Orthodox fasting versus time‐restricted eating on plasma irisin concentrations among overweight metabolically healthy individuals. Nutrients. 2021;13:1071. 33806150 10.3390/nu13041071 PMC8064431

[2] Karras SN , Koufakis T , Adamidou L , et al. Similar late effects of a 7‐week orthodox religious fasting and a time restricted eating pattern on anthropometric and metabolic profiles of overweight adults. Int J Food Sci Nutr. 2021;72:248‐258. 32605472 10.1080/09637486.2020.1787959

[3] Joaquim L , Faria A , Loureiro H , Matafome P . Benefits, mechanisms, and risks of intermittent fasting in metabolic syndrome and type 2 diabetes. J Physiol Biochem. 2022;78:295‐305. 34985730 10.1007/s13105-021-00839-4

[4] van Loon LJ , Kruijshoop M , Menheere PP , Wagenmakers AJ , Saris WH , Keizer HA . Amino acid ingestion strongly enhances insulin secretion in patients with long‐term type 2 diabetes. Diabetes Care. 2003;26:625‐630. 12610012 10.2337/diacare.26.3.625

[5] Gannon MC , Nuttall FQ . Amino acid ingestion and glucose metabolism‐‐a review. IUBMB Life. 2010;62:660‐668. 20882645 10.1002/iub.375

[6] Food Processor Analysis Software. 2018. Accessed on July 2, 2022. https://www.esha.com/products/food-processor/.

[7] Lindgren O , Pacini G , Tura A , Holst JJ , Deacon CF , Ahrén B . Incretin effect after oral amino acid ingestion in humans. J Clin Endocrinol Metab. 2015;100:1172‐1176. 25490278 10.1210/jc.2014-3865

[8] Zhou Z , Yin H , Guo Y , et al. A fifty percent leucine‐restricted diet reduces fat mass and improves glucose regulation. Nutr Metab (Lond). 2021;18:34. 33771176 10.1186/s12986-021-00564-1 PMC7995702

[9] Karusheva Y , Koessler T , Strassburger K , et al. Short‐term dietary reduction of branched‐chain amino acids reduces meal‐induced insulin secretion and modifies microbiome composition in type 2 diabetes: a randomized controlled crossover trial. Am J Clin Nutr. 2019;110:1098‐1107. 31667519 10.1093/ajcn/nqz191 PMC6821637

